# Straight A’s: protein acylation in the *S-*acylation and autophagic degradation of NOD-like receptors

**DOI:** 10.1042/BST20253026

**Published:** 2025-07-04

**Authors:** Noah R. Martin, Gregory D. Fairn

**Affiliations:** 1 Department of Pathology, Faculty of Medicine, Dalhousie University, Halifax, Nova Scotia, Canada; 2 Department of Biochemistry and Molecular Biology, Faculty of Medicine, Dalhousie University, Halifax, Nova Scotia, Canada

**Keywords:** inflammation, NF-κB, NLRP3, NOD2, *S*-acylation, ZDHHC

## Abstract

Over the past decade, *S*-acylation has emerged as a crucial regulator of several innate immune signaling pathways, with new insights continually being gained. *S*-acylation, a reversible post-translational modification, involves the attachment of fatty acyl chains to cysteine residues, influencing protein localization, function, and stability. In this mini-review, we examine the accumulating evidence of the role of *S*-acylation in regulating nucleotide oligomerization domain (NOD)-like receptors. NOD-like receptor subfamily P3 (NLRP3), a key player in inflammasome formation, undergoes *S*-acylation at specific cysteine residues, which are essential for its localization to the *trans*-Golgi network and other organelles. Various zinc finger Asp-His-His-Cys motif-containing (zDHHC) enzymes mediate this modification, with zDHHC5 being particularly important for activation and the ability of NLRP3 to interact with never in mitosis gene A (NIMA)-related protein kinase 7 (NEK7), promoting inflammasome assembly, caspase-1 activation, and pyroptosis. Alternatively, *S*-acylation by zDHHC12 targets NLRP3 for chaperone-mediated autophagy, preventing excessive inflammation. NOD2, another NLR, requires *S*-acylation for membrane localization and effective signaling via the NF-κB and mitogen-activated protein kinase pathways in response to peptidoglycan components. Dysregulation of *S*-acylation in NOD2 is associated with Crohn’s Disease (hypo-acylated) and Blau syndrome/early-onset sarcoidosis (hyper-acylated). Soluble NOD2 lacking *S*-acylation is ubiquitinated and eliminated by the autophagic pathway. This review highlights the significance of understanding the *S*-acylation cycle and its regulatory mechanisms in developing potential therapeutic interventions for related inflammatory diseases. We also discuss unresolved questions regarding the *S*-acylation of NOD2 and NLRP3, as well as the regulation of *S*-acylation in general.

## NOD-like receptor family

The innate immune system is our most ancient defense against microorganisms, initiating a nonspecific response to rid the body of these assailants [1]. Pattern recognition receptors (PRRs) are critical proteins expressed in immune and epithelial cells, two cell types that mediate much of our interactions with the outside world [2]. PRRs initiate inflammatory signaling upon binding their ligand, typically derived from the repeating polymers of the cell wall of bacteria and fungi, referred to as pathogen-associated molecular patterns (PAMPs) or microbe-associated molecular patterns (MAMPs) [2]. Furthermore, some PRRs can detect host-derived ligands termed damage-associated molecular patterns (DAMPs) [3]. Following ligand engagement, PRRs typically interact with additional proteins that generate signal transduction pathways, such as NF-κB, or the activation of caspases [2]. Ultimately, activating these pathways can lead to the release of cytokines and other modulators, alerting the immune cells to the threat, and, in some cases, this can result in cell death [4].

The nucleotide-binding oligomerization domain-like receptor (NOD-like receptor; NLR) family comprises a subset of mammalian PRRs [5]. NLRs play a crucial role in detecting microbial infiltration and cellular stress, as well as initiating inflammatory signaling. NLRs typically consist of three main domains: an effector domain at the N-terminus of the protein allows for interactions with signaling mediators [6]. A central neuronal apoptosis inhibitor protein (NAIP), CIITA, HET-E, and TP-1 (NACHT) domain is found in all NLRs, named for the proteins in which it was initially discovered [6]. The NACHT is a nucleoside triphosphatase domain that allows for the binding and hydrolysis of nucleoside triphosphates (ATP/GTP), facilitating conformational changes and protein oligomerization [6]. Sequence alignment of the central NACHT domain is consistent throughout the NLR family with slight variations [7]. Finally, a region of leucine-rich repeats (LRR) is found at the C-terminal end of the protein; these repeats are responsible for binding ligands typically derived from microbial invaders. This results in a conformational change in the protein, which is required for self-oligomerization, downstream protein–protein interactions, and signal propagation [6].

**Figure 1:**
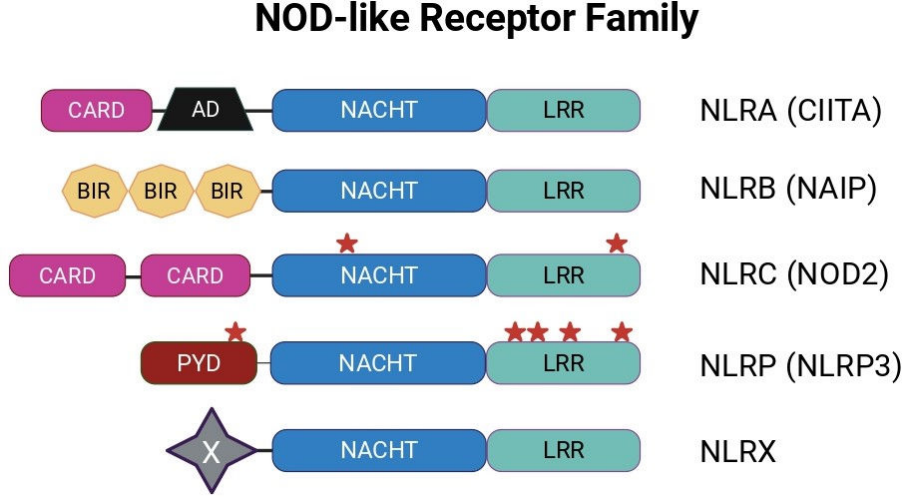
NOD-like receptor family members. The structure and organization of NLR family members are shown here. All human NLRs share a central NACHT domain and LRR region at the C-termini of the protein, as well as a variable N-terminal effector domain. The LRR is responsible for ligand sensing, and the NACHT allows for nucleotide binding, hydrolysis, and oligomerization with other NLRs. At the same time, the N-terminal effector domain dictates interactions with signaling mediators and the specificity of activated signaling pathways. The relative locations of the S-acylation sites on NOD2 and NLRP3 are denoted with red stars.

NLRs can be further divided into five subfamilies based on the identity of their respective N-terminal domains (Figure 1). NLRA, A for an acidic-transactivating domain, includes Class II major histocompatibility complex and transactivator, CIITA. NLRB, B for baculovirus inhibitor repeat domain, identified based on homology to the baculovirus inhibitor of apoptosis proteins. NLRC, C for caspase-activation recruitment domain (CARD), present in NOD1 and NOD2, which use this domain to interact with and activate receptor-interacting serine-threonine kinase 2 (RIP K2). NLRP, P stands for a pyrin domain and is critical for protein–protein interactions in inflammasome formation. Finally, NLRX, where X signifies a lack of homology to the other domains [8].

## Protein *S*-acylation and the zDHHC family

Many NLR family members undergo post-translational modification, with *S*-acylation being critical for association with cellular membranes. *S*-acylation is a reversible modification for many proteins in which a fatty acyl chain is covalently attached to a cysteine residue on a protein via a thioester linkage [9]. For a few proteins, this can occur spontaneously (auto-*S*-acylation). In contrast, for most others, this modification is performed by zinc finger Asp-His-His-Cys motif-containing (zDHHC) protein acyltransferases [9,10]. Individual members of the zDHHC family are found in the endoplasmic reticulum, Golgi apparatus, plasma membrane, the nuclear membrane, endosomes, and secretory vesicles [11–13]. Mechanistically, zDHHC proteins are auto-acylated. In this transacylation reaction, the acyl chain from acyl-CoA is transferred to the Cys residue in the zDHHC active site, forming an acyl-zDHHC intermediate, which is then transferred to the protein substrate [14]. The precise mechanism by which this occurs at the molecular level and the contribution of neighboring amino acids remains unclear. The mechanism by which zDHHCs recognize their substrates is also poorly understood. However, in some cases, such as zDHHC13 and zDHHC17, it has been reported that the N-terminal ankyrin repeats play a role in substrate recognition [15]. Regardless, the addition of acyl groups to proteins or protein domains increases their hydrophobicity, thereby promoting their partitioning into membranes [16]. As a result, *S*-acylation can influence many properties of a protein, including localization, sorting, trafficking between organelles, stability, and catalysis [9].

The removal of the acyl chain is catalyzed by a growing number of acyl thioesterases, many of whom contain the α/β hydrolase fold or domain [17,18]. The α/β hydrolase domain (ABHD) is common to many hydrolytic enzymes with diverse functions, including proteases, lipases, esterases, and epoxide hydrolases. One challenge for the field is that many ABHD enzymes have multiple reported substrates, including lipids and *S-*acylated proteins. Currently, the best-characterized ABHD enzymes that act as acyl protein thioesterases are acyl protein thioesterase 1 (APT1) and APT2, ABHD17 isoforms, and palmitoyl-protein thioesterase 1. However, other unique deacylating enzymes have been characterized, such as ceroid lipofuscinosis, neuronal 5 (CLN5), which uses a cysteine nucleophile rather than a serine [19], or the case of the antioxidant PRDX6, which utilizes glutathione to deacylate its substrates [20]. The acylation-deacylation cycle varies from fast (~5 min), as seen with N-Ras [21], to virtually no turnover, as reported for caveolin-1 [22]. *S*-acylation dynamics vary widely across the *S-*acylome/palmitoylome, and proteins with a quick turnover have been linked to carefully regulated pathways whose perturbation leads to disease [23]. The controlled and regulated *S*-acylation and deacylation of proteins offer yet another way to fine-tune a protein’s function. Indeed, some zDHHC proteins, such as zDHHC5, are themselves *S*-acylated and activated by other zDHHCs in an *S*-acylation cascade. This adds another layer of control of *S*-acylation and is vital to consider, especially when interpreting data generated from the overexpression of zDHHC proteins [24]. Our collective understanding of this mode of regulation is in its infancy. However, there are clear examples of the *S*-acylation cycle being a point of control for proteins, including for the NLRs [25,26].

## A short primer on the NLRP3 structure, function, and inflammasome formation

The activation of the NLRP3 inflammasome leads to the conversion of pro-caspase-1 into its cleaved and mature form. Active caspase-1 can then process precursor forms of the cytokines IL-1β and IL-18 [27]. Additionally, caspase-1 can initiate a form of inflammatory programmed cell death termed pyroptosis through the cleavage and activation of gasdermin-D. The concerted actions of gasdermin-D and ninjurin-1 (NINJ1) permeabilize the plasma membrane, allowing cytokines and other cytosolic contents to spill out of the dying cell and stimulate an inflammatory response [28].

NLRP3 signaling can be divided into three discrete steps: priming, activation, and termination [29]. Priming requires the activation of the NF-κB pathway by other PRRs, leading to the up-regulation and production of NLRP3 [30]. Following localization, the activation of the NLRP3 inflammasome requires the sensing of PAMPs and DAMPs [31]. PAMPs activating NLRP3 include bacterial toxins, viral RNA, and fungal glucans [31]. DAMPs recognized by NLRP3 include cholesterol and urate crystals, oxidized mitochondrial DNA, ceramides, silica, and alum, which can be derived from either self or foreign origin [28]. It should be noted that while some studies suggest that localization occurs before NLRP3 activation, others indicate that activation can occur before localization [32]; further research is needed to determine the exact sequence of events and their context dependence.

The NLRP3 inflammasome is a multiprotein complex comprising NLRP3, an adapter protein apoptosis-associated speck-like protein containing a CARD (ASC), caspase-1, and NIMA-related protein kinase 7 (NEK7) [33]. Following stimulation of NLRP3, self-oligomerization occurs via the NACHT domain, resulting in the formation of a cage structure. The association of NLRP3 with NEK7 is critical for inflammasome signaling; the interaction of NEK7 leads to the opening of the inactivated NLRP3 cage structure, allowing for the recruitment of ASC. However, recent studies have shown that it may be possible to activate the NLRP3 inflammasome without the presence of NEK7, such as via priming by IKKβ [34]. Thus, additional research is required to fully elucidate the dispensability of NEK7 in the activation of the NLRP3 inflammasome. ASC acts as an adaptor to recruit pro-caspase-1, leading to self-cleavage and activation, allowing caspase-1-dependent cleavage of proinflammatory cytokines (pro-IL-1β and pro-IL-18), as well as gasdermin D. The cleaved, N-terminal domain of gasdermin D, which is also *S*-acylated, assembles in the plasma membrane, resulting in the activation of NINJ-1, another pore-forming assembly, which ultimately allows cytokines and other cytosolic components to escape the cell during pyroptosis [28]. Recent studies have identified *S*-acylation as a critical regulator of NLRP3, impacting its localization, activation, and chaperone-mediated autophagy.

## *S*-acylation of NLRP3 regulates the inflammasome

### Localization

**Figure 2:**
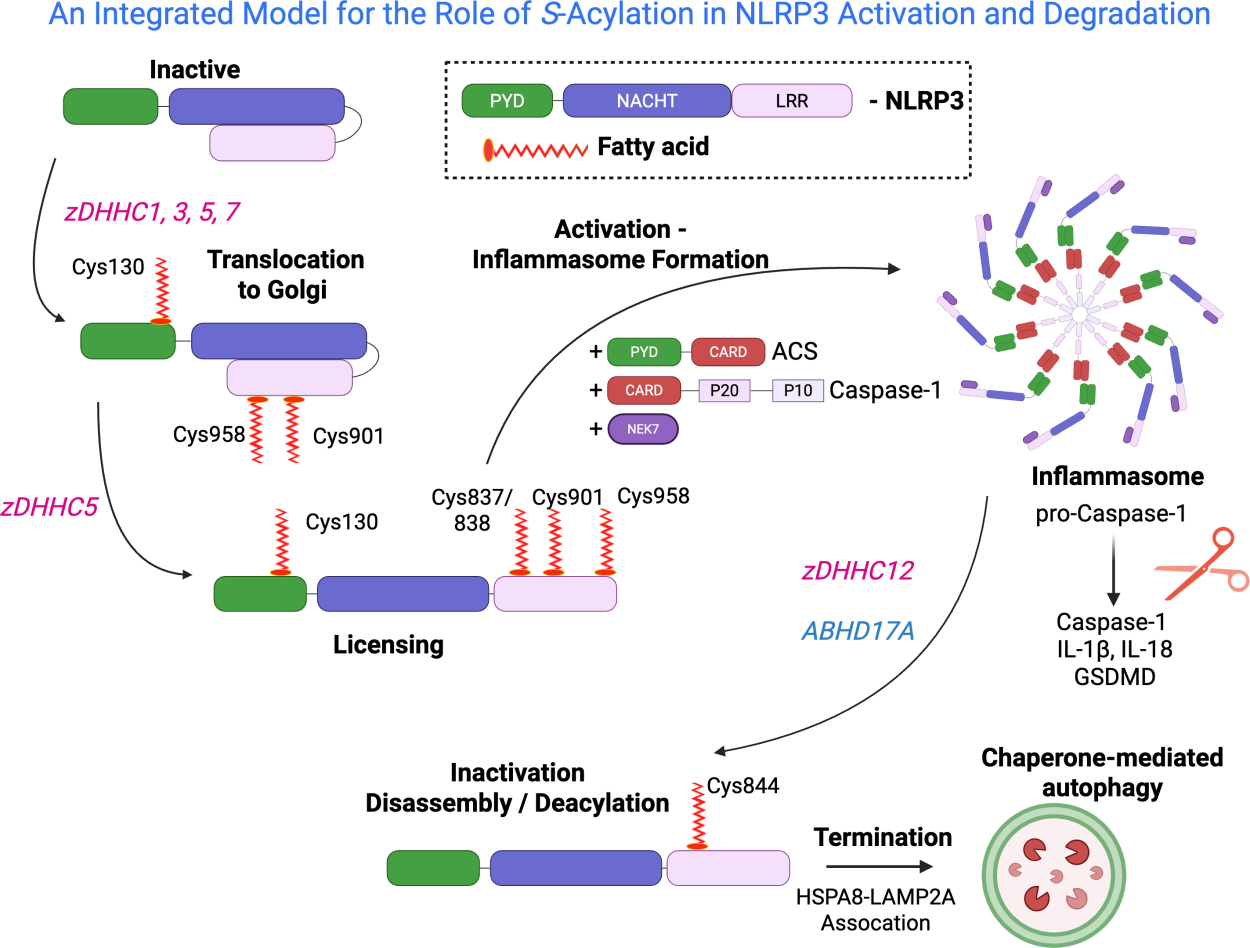
Proposed model of the integrated control of the NLRP3 Inflammasome by *S-*acylation. *S*-acylation of NLRP3 by zDHHC3/7 promotes localization to the TGN and possibly other organelles. Further *S*-acylation by zDHHC5 and NEK7 binding licenses NLRP3, allowing for ligand sensing and oligomerization, resulting in the formation of the inflammasome and activation of downstream effectors. Finally, *S*-acylation by zDHHC12 acts as an emergency brake to halt NLRP3-induced signaling. This specific *S*-acylation leads to the interaction of NLRP3 with HSPA8-LAMP2A, resulting in the lysosomal degradation of the protein by chaperone-mediated autophagy.

Following priming, NLRP3 must localize to the surface of organelles to ultimately facilitate the interactions with ASC and NEK7 [31]. The identity of this organelle has been debated in recent years, and initial reports have demonstrated that a dispersed *trans*-Golgi network (dTGN) was the key mediator [35]. Lysosomes have recently been reported as supporting inflammasome activation [36]. In 2024, Lin and colleagues reported that *S*-acylation of Cys130 is crucial for localization to the TGN and subsequent trafficking to the microtubule organizing center (MTOC) for inflammasome formation and signaling; this *S*-acylation is catalyzed by zDHHC7 [37]. Note, for simplicity, we will use the residue number for the human NLRP3 since work has been done with both mouse and human. Inhibition or knockout of zDHHC7 in mouse bone marrow-derived macrophages results in the impairment of NLRP3 localization and diminishes NLRP3 signaling [37]. Similarly, Chen and colleagues report similar findings, stating that S-acylation of Cys130 and Cys958 drives the localization of NLRP3, which is required for subsequent inflammasome signaling [38]. However, rather than zDHHC7, the authors report that the activity of zDHHC1 acylates NLRP3 on these Cys residues [38]. In this study, the inactivation of zDHHC1 partly abolished the *S*-acylation of NLRP3, indicating that multiple zDHHC enzymes may contribute to the *S*-acylation of NLRP3 [38]. A report by Penden and colleagues further supports this notion, finding that *S*-acylation of Cys130 is mediated by zDHHC3 and zDHHC7, and that overexpression of either transferase increases the TGN localization of NLRP3 [39]. However, it is important to note that zDHHCs are often expressed at low levels, and overexpression of these enzymes may lead to promiscuity in substrate selection, as well as other nonphysiological findings. Therefore, results generated from zDHHC overexpression should be cautiously interpreted and complemented by other techniques [40]. Nunez and colleagues report that Cys130 *S*-acylation by zDHHC5 regulates TGN localization and inflammasome signaling [41]. Finally, Anand and colleagues outline the importance of *S*-acylation in the localization and activation of NLRP3 inflammasome signaling by inhibiting fatty acid synthesis in mice, resulting in a significant abrogation of NLRP3 signaling. Additionally, predicted *S*-acylation sites were mutated to alanine, and these mutants were assessed for *S*-acylation, identifying Cys901 as the residue crucial for initiating the signaling pathway. Through the inhibition of fatty acid synthesis and the broad-spectrum *S*-acylation inhibitor 2-bromopalmitate (2 BP), the authors demonstrated the necessity of Cys901 for the TGN localization of NLRP3 by isolating these dispersed vesicles and probing for NLRP3, showing that disruption of *S*-acylation prevents TGN localization. It should be noted that 2 BP treatment is cytotoxic and nonspecific in its inhibition of *S-*acylation and is known to target other proteins such as acyl thioesterases. This should be kept in mind when interpreting the results of experiments utilizing 2 BP [42]. Together, these studies highlight the importance of this PTM and underscore the challenges in monitoring *S*-acylation, as many substrates and zDHHC enzymes exhibit promiscuity (Figure 2).

### Activation

The activation of the NLRP3 inflammasome refers to the oligomerization and formation of the inflammasome, followed by the caspase-1-dependent maturation and release of proinflammatory cytokines [43]. Once localized to the TGN, NLRP3 can detect MAMPs and DAMPs, leading to the dispersal of the TGN and vesicular trafficking to the MTOC, where inflammasome formation can occur through interaction with ASC and caspase-1 [28]. NLRP3 oligomerization forms a closed, inactive, double-ring cage-like structure on the membrane. In this conformation, the pyrin domains and leucine-rich repeats are inaccessible, preventing activation. Pei and colleagues report that NLRP3 is *S-*acylated at residues Cys837 and Cys838 by zDHHC5. This modification causes a conformational change in the NLRP3 leucine-rich repeat region, promoting interaction with NEK7 and ‘licensing’ [44]. The resulting opening of the double-ring, cage-like NLRP3 structure enables interactions with ASC and pro-caspase-1, facilitating inflammasome formation. Overexpression of zDHHC2, 3, 5, and 7 led to an increase in NLRP3 *S*-acylation; however, when the expression of these zDHHCs was silenced, only the removal of zDHHC5 significantly reduced the *S*-acylation of NLRP3, leading the authors to conclude that zDHHC5 is the major transferase enzyme involved in NLRP3 *S*-acylation [45]. Additionally, the knockout of zDHHC5 resulted in diminished inflammasome signaling, shown by a reduction in caspase-1 activation, IL-1β and IL-18 release, and gasdermin-D cleavage. Finally, mutation of Cys837/838 to alanine had similar effects to those of zDHHC5 knockout, reducing activation and signaling. However, these alanine mutations did not affect the TGN localization of NLRP3, nor did they affect trafficking to the MTOC. The authors also report that ABHD17 isoform A can deacylate NLRP3 at positions Cys837/838, suggesting that this modification may be reversible, as inactivation of ABHD17A increases NLRP3 signaling [45] (Figure 2). This study demonstrates that specific *S*-acylation events on Cys837/838 are critical switches to aid in the licensing of NLRP3 together with NEK7 binding. The ability of ABHD17A to inactivate the licensed NLRP3 may also serve as a vital checkpoint to limit unneeded signaling.

## *S*-acylation and chaperone-mediated autophagy (CMA) as an emergency brake for the NLRP3 inflammasome

Degradation of the NLRP3 may represent a critical mode of regulation against aberrant activation of the NLRP3 inflammasome. NLRP3 is a target of the chaperone-mediated autophagy (CMA) pathway, leading to the selective lysosomal degradation of the protein [46]. CMA is driven by the association of the protein destined for degradation with heat shock 70 kDa protein 8 (HSPA8). HSPA8 recognizes lysine-phenylalanine-glutamic acid-arginine-glutamine (KFERQ)-like motifs on proteins and delivers these cargoes to lysosomal-associated membrane protein 2A (LAMP2A) on the lysosome, resulting in the lysosomal degradation of the cargo protein [47]. Again, *S*-acylation is a critical PTM that has been shown to regulate the CMA of NLRP3 [46,48]. In two studies, Cui et al. demonstrate that *the S*-acylation of NLRP3 on Cys844 by zDHHC12 targets NLRP3 for CMA, resulting in increased stability of NLRP3 in zDHHC12 knockout cells compared with those in the wildtype (WT) cells. The activation of NLRP3 is shown to increase the Cys844 *S*-acylation of NLRP3, as well as the expression of the acylating enzyme zDHHC12, in the later stages of activation. This serves to dampen the NLRP3 response and prevent excessive signaling and pyroptosis. Knockout of zDHHC12 has been shown to reduce the interaction between NLRP3, HSPA8, and LAMP2A compared with WT cells. Upon structural analysis, the authors discovered that the Cys844 site is in proximity to the KFERQ-like motif of NLRP3 and postulate that this *S*-acylation may confer a conformational change to NLRP3, making the motif more accessible to HSPA8, which explains the reduction in degradation in zDHHC12 knockout cells. To solidify the role of the CMA via the lysosome as the mechanism responsible for NLRP3 degradation, the authors employed proteasome and lysosome inhibitors, finding that the lysosomal inhibitors block NLRP3 degradation. In contrast, proteasome inhibitors do not [46,48].

## *S*-acylation of NOD1 and NOD2 is required for peptidoglycan sensing

**Figure 3:**
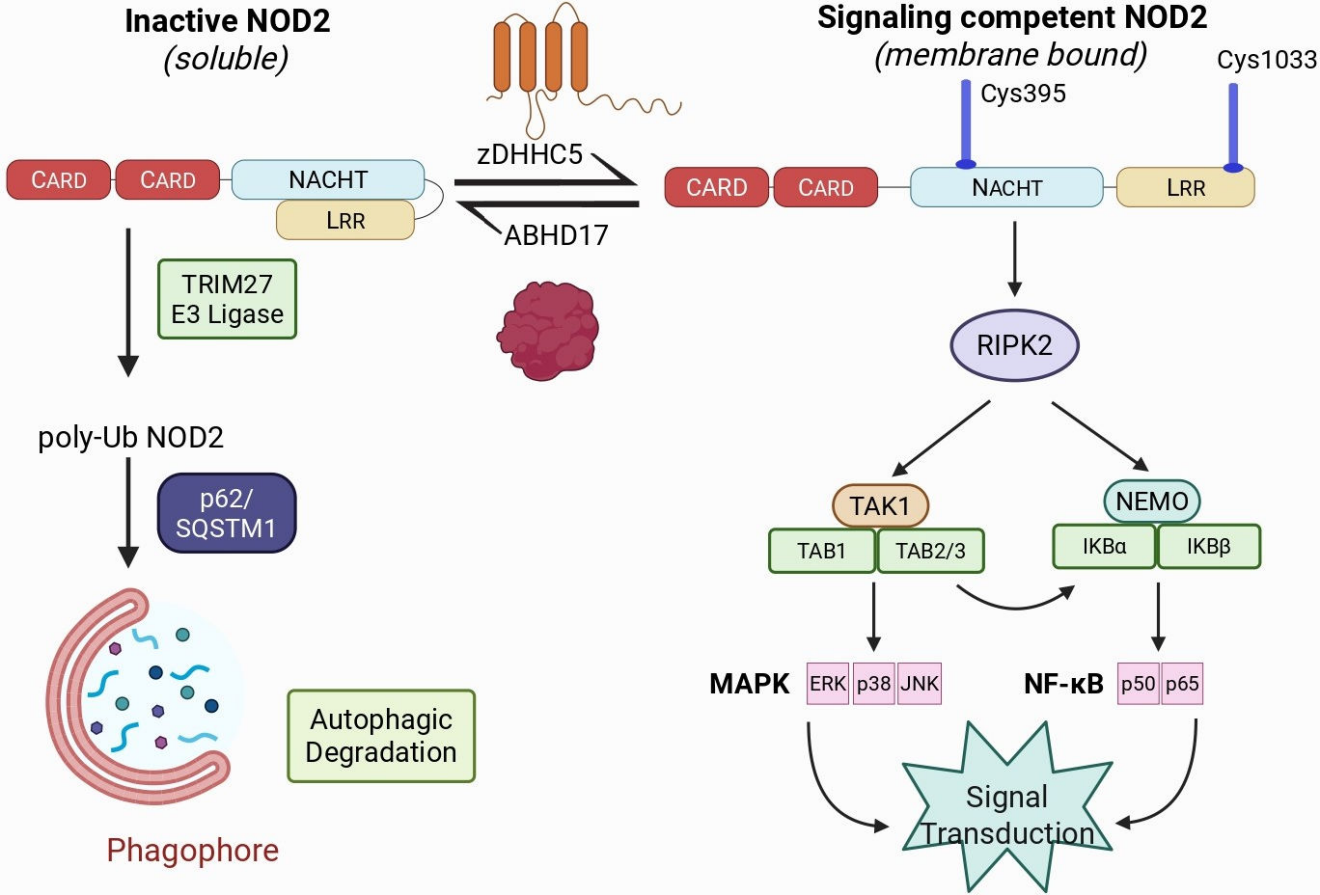
NOD2 *S-*acylation affects stability and signaling. Inactive NOD2 is acted upon by zDHHC5, resulting in NOD2 being *S*-acylated at two positions. *S*-acylation is required for targeting to the plasma membrane, endosomes, and phagosomes and is necessary for oligomerization. Oligomerization enables interaction with RIPK2 and the activation of the NF-κB and MAPK signaling pathways. *S*-acylated NOD2 can be deacylated by the three members of the ABHD17 family (ABHD17A, ABHD17B, and ABHD17C), thereby returning NOD2 to its soluble and inactive state. In the absence of *S*-acylation, NOD2 can be ubiquitinated, leading to interactions with the SQSTM1/p62 autophagic cargo receptor, resulting in its removal by autophagy.

NOD1 and NOD2 respond to components of bacterial peptidoglycan and signal via the NF-κB and mitogen-activated protein kinase (MAPK) pathways; the NOD1 ligand is γ-D-glutamyl-*meso*-diaminopimelic acid (iE-DAP) [49] and muramyl dipeptide (MDP) is the ligand recognized by NOD2 [10]. It is proposed that upon the binding of the NOD ligand (iE-DAP/MDP) to the LRR region, a conformational change opens the protein and unmasks the activity of the Walker-A and Walker-B boxes, allowing for ATP binding and hydrolysis, leading to self-dimerization and further assembly into the functional NOD signaling unit, the NODosome [50]. However, an alternative explanation is that the binding of ATP to the nucleotide binding domain (NBD) triggers the conformational change, and ligand binding acts as a mechanism to prolong the ‘open’ NOD conformation [51]. Regardless, the formation of the NODosome enables the orientation of the NOD CARD domains, allowing for hetero-CARD complexation with the CARD domain of RIPK2, which is responsible for interacting with intermediates of the NF-κB and MAPK pathways, thereby facilitating signal propagation (Figure 3).

Membrane localization, either to the plasma membrane or endosomal membranes, is necessary for response to peptidoglycan and RIPK2-dependent signaling [10]. However, NOD proteins lack transmembrane domains or other classical lipid-binding motifs, which would promote this membrane localization. Instead, *S-*acylation at two positions in NOD2 and three positions in NOD1 localizes the protein to the plasma membrane and endosomes. zDHHC5 is responsible for the *S*-acylation of NOD1 and NOD2; NOD1 is *S*-acylated at positions C558, 567, and 952, and NOD2 at sites C395 and 1033 [10]. For NOD1, *S-*acylation occurs just before and within the LRR domain. *S-*acylation of NOD2 occurs within the NBD and LRR domains. *S-*acylation of NOD1 and NOD2 is reversible, and the activity of ABHD17 isoforms removes the fatty acyl chains [18]. Thus, the interplay between these two enzymes allows for fine-tuning of the membrane-localized and signaling-capable pool of NODs. Upon membrane localization and oligomerization to form the NODosome, interactions with RIPK2 via the CARD domains lead to the polymerization and K63-linked ubiquitination of RIPK2. This modification enables recruitment and interaction with mediators of the NF-κB and MAPK pathways, including the NF-κB essential modulator subunit, as well as transforming growth factor Β-Activated kinase 1 (TAK1) and TAK1 binding protein 1. These interactions ultimately lead to the ubiquitination and proteasomal degradation of inhibitor subunits of the NF-κB pathway, as well as the phosphorylation and nuclear translocation of transcription factors such as p38, p50, p65, extracellular signal-regulated protein kinase, and c-Jun N-terminal kinase. These transcription factors result in the expression of pro-inflammatory cytokines and chemokines, such as GM-CSF and IL-8, and anti-microbial peptides such as β-defensin 2 [52–54].

As the *S*-acylation of NOD2 controls signaling, perturbation of this modification can lead to defective signaling and disease. NOD2 variants that impair signaling ability are associated with Crohn’s disease, leading to dysbiosis in the gastrointestinal tract. Some of these variants exhibit a reduced degree of *S-*acylation compared with the WT NOD2 [10]. In contrast, others display an inability to recognize MDP or defects in the CARD domains, which prevents downstream signaling. Recent evidence suggests that inhibiting the ABHD17A, B, and C isoforms can rescue the *S-*acylation and signaling ability of some, but not all, Crohn’s disease-associated NOD2 variants. This suggests that some mutants may be poor substrates for the zDHHC5 enzyme, while others may not be recognized at all.

Conversely, the excessive *S-*acylation of NOD2 can lead to hyperactive signaling in the absence of stimuli. Blau syndrome (BS) is an autoinflammatory disease of the skin, eyes, and joints underpinned by excessively signaling NOD2 variants. NOD2 variants, such as C495Y, are excessively *S*-acylated, localized to the membrane, and exhibit hyperactive signaling without stimulation, leading to the symptoms of BS [55]. As excessive NOD2-induced signaling is detrimental, there must be a mechanism to regulate NOD2 expression and signaling. NOD2 is known to undergo autophagic degradation via interactions with SQSTM1/p62, an autophagic cargo receptor, and proteasomal degradation [56]. The E3 ligase tripeptide motif-containing 27 has been observed to co-immunoprecipitate with NOD2 and catalyze the K48-linked polyubiquitination of NOD2, leading to its degradation by the 26S proteasome [57]. *S*-acylated NOD2 appears to be protected from autophagic degradation [56]. For instance, treatment with 2 BP, an *S-*acylation inhibitor, resulted in the loss of NOD2 from the plasma membrane and a reduction in NOD2 protein abundance. Using autophagy inhibitors or cell lines with defects in autophagy prevented the degradation of deacylated NOD2, whereas proteasome inhibitors did not, indicating that the degradation of deacylated NOD2 is an autophagic process [56]. The KO of SQSTM1/p62 abolished the autophagic degradation of NOD2, showing that this autophagic cargo receptor is responsible for the degradation of NOD2 [56]. Thus, the *S*-acylation cycle and autophagy are critical mechanisms for regulating the abundance and activation state of NOD2, as degradation acts to prevent excessive NOD2 signaling by degrading NOD2 that is not *S*-acylated. The relevant contribution of the proteasome and autophagy in regulating NOD2 abundance awaits further interrogation in relevant cell lines.

## Conclusions and future directions

Although first discovered in 1979, the study of *S*-acylation as a post-translational modification was initially slow. Fortunately, over the last two decades, the development of methods such as acyl resin-assisted capture, acyl biotin exchange, and bioorthogonal chemistry has enabled the replacement of radioactive lipids as a readout of protein *S*-acylation. At its core, *S*-acylation and de-*S-*acylation are critical switches that influence protein localization, interactions, and function, as illustrated here by NLRP3 and NOD2. The *S*-acylation of NOD1 and NOD2 is relatively straightforward: defined residues and a single zDHHC5. However, NOD2 is also reported to be activated by other ligands, including sphingosine 1-phosphate and single-stranded RNA [58,59]. Whether these pathways require zDHHC5, or *S*-acylation in general, is currently unknown. Research will be required to determine if these RIPK2-independent pathways require the presence of the acyl chain on the protein.

The regulation of NLRP3 by *S*-acylation is complex and appears to have temporally discrete roles. In Figure 2, we propose an integrated model of NLRP3 inflammasome regulation by *S*-acylation. The collective studies highlight the involvement of many residues and zDHHC enzymes. Many of the studies seem incongruent. However, it is worth considering that differences in cell types and species, as well as the relative abundance of individual zDHHC enzymes, may account for some of these differences. It is well established in the literature that some zDHHC enzymes recognize a wide range of substrates, while others appear to be more promiscuous. The subcellular localization of zDHHCs involved in the *S*-acylation of NLRP3, which regulates proper localization, activation, and degradation, is challenging to establish due to varying claims regarding the participating enzymes. Further research into the zDHHCs responsible for these modifications will be required before concluding on the localization of each *S-*palmitoylating enzyme. We suspect that future studies will attempt to delineate this in greater detail, while also resolving the temporal order of events. We also wonder if there is a need for additional acyl protein thioesterases to remove some of the acyl chains, allowing for subsequent modifications or CMA to occur. Given the importance of the NLRP3 inflammasome, we are confident that these experiments are ongoing.

While focusing on regulating NLR proteins by *S*-acylation, this mini-review did not consider the regulation of the acyltransferases conferring this lipid modification. This is mainly due to the fact that, in the case of NLRP3 and NOD2, those details remain to be explored. Indeed, except for a few specific instances, little remains known about how zDHHCs are regulated beyond subcellular localization [60], and the same holds true for the regulation of deacylating enzymes. Future studies will be required to determine how the zDHHC enzymes recognize their substrates and whether their activity is regulated by post-translational modifications (such as phosphorylation, *S*-acylation, and glycosylation), the binding of inhibitory proteins, or other mechanisms.

In general, zDHHC proteins are subject to the influence of many standard regulatory mechanisms. This includes transcriptional control and translation, and protein–protein interactions with accessory proteins. At the transcriptional level, zDHHCs have tissue-specific expression patterns and, in addition, can be stabilized or re-localized by an increase in the concentration of their substrates or up-regulated in response to stimuli detected by the cell [61,62]. The zDHHC accessory protein interaction is known to stabilize and promote the activity of the enzyme, as exemplified by zDHHC5 and Golga7/Golga7b. These proteins form a complex that acts to stabilize zDHHC5 at the plasma membrane and prevent its endocytosis at least in some cell types [63,64]. Thus, Golga7a and 7b would conceivably be required for the *S-*acylation of NOD2; however, this remains to be demonstrated experimentally.

Post-translational modifications, such as phosphorylation and *S*-acylation, play a role in the localization and activity of zDHHCs. Notably, for zDHHC5, it has been found that phosphorylation acts as a brake, reducing activity and membrane localization, while promoting degradation in response to heightened neuronal activity [65]. Lastly, *S-*acylation of zDHHC5 by zDHHC20 enhances the plasma membrane localization and, consequently, the activity of zDHHC5. zDHHC5 is acylated within its C-terminal tail, which helps pin the enzyme to the membrane and prevents mislocalization. This enables the proper spatial organization of zDHHC5, which is essential for the effective *S*-acylation of its substrates [66].

With further study on this topic, the intricacies of precisely controlling immune signaling through *S*-acylated proteins will be revealed, enabling the development of methods to regulate and utilize these signaling pathways.

**Perspectives**

- The nucleotide oligomerization domain (NOD)-like receptor family is a critical mediator of inflammation. NOD1, NOD2, and NOD-like receptor subfamily P3 (NLRP3) require post-translational *S*-acylation to be functional.

- The ability to manipulate the *S*-acylation of NOD2 and NLRP3 may prove beneficial in Crohn’s disease and sepsis, respectively

- The NOD-like receptor family has many members beyond NOD1, NOD2, and NLRP3. This raises the possibility that additional family members may also be regulated by *S*-acylation.

## Open Access

This article has been published open access under our Subscribe to Open programme, made possible through the support of our subscribing institutions, learn more here: https://portlandpress.com/pages/open_access_options_and_prices#conditional

## Abbreviations:

ABHD: α/β hydrolase domain
ASC: apoptosis-associated speck-like protein containing a CARD
BP2 BP: 2-bromopalmitate
BS: blau syndrome
CARD: caspase activation recruitment domain
CIITA: class II, major histocompatibility complex transactivator
CMA: chaperone mediated autophagy
DAMP: damage associated molecular pattern
GM-CSF: granulocyte-macrophage colony-stimulating factor
HSPA8: heat shock 70 kDa protein 8
IKKβ: inhibitor of nuclear factor kappa-B kinase subunit beta
IL-8: Interleukin-8
IL-18: interleukin-18
IL-1β: interleukin-1 beta
KFERQ: lysine-phenylalanine-glutamic acid-arginine-glutamine
LAMP2A: lysosomal-associated membrane protein 2A
LRR: leucine-rich repeats
MAMP: microbe associated molecular pattern
MAPK: mitogen-activated protein kinase
MDP: muramyl dipeptide
MTOC: microtubule-organizing center
NACHT: neuronal apoptosis inhibitor protein (NAIP), CIITA, HET-E and TP-1 domain
NBD: nucleotide binding domain
NEK7: NIMA-related kinase 7
NIMA: never in mitosis gene a
NINJ-1: Ninjurin-1
NLR: NOD-like receptor
NLRA: NLR subfamily A
NLRB: NLR subfamily B
NLRC: NLR subfamily C
NLRP: NLR subfamily P
NLRX: NLR subfamily X
NOD: nucleotide oligomerization domain
N-RAS: neuroblastoma RAS viral oncogene homolog
PAMP: pathogen associated molecular pattern
PPT: palmitoyl-protein thioesterase
PRR: pattern recognition receptor
PTM: post-translational modification
RIPK2: receptor-interacting serine/threonine-protein kinase 2
SQSTM1: sequestosome 1
TAK1: transforming growth factor beta activated kinase 1
TGN: trans-Golgi network
TLR4: toll-like receptor 4
WHD: winged helical domain
WT: wildtype
dTGN: dispersed trans-Golgi network
iE-DAP: D-γ-glutamyl-meso-DAP dipeptide
ssRNA: single-stranded ribonucleic acid
zDHHC: zinc finger Asp-His-His-Cys motif-containing
